# A case of complete atrioventricular block with extremely high blood concentration of azelnidipine

**DOI:** 10.1186/s40780-021-00230-x

**Published:** 2021-12-01

**Authors:** Naohito Ide, Ayaka Mochizuki, Yoshiyuki Kagawa, Masaharu Ito

**Affiliations:** 1 Department of Pharmacy, Chutoen General Medical Center, 1-1 Shobugaike, Kakegawa, Shizuoka, 436-8555 Japan; 2 Laboratory of Clinical Pharmaceutics, School of Pharmaceutical Sciences, University of Shizuoka, 52-1 Yada, Suruga-ku, Shizuoka, 422-8526 Japan

**Keywords:** Azelnidipine, Adverse events, Calcium channel blocker, Complete atrioventricular block, Drug interaction, Simvastatin

## Abstract

**Background:**

Azelnidipine, a dihydropyridine calcium channel blocker (CCB), has less adverse effects (e.g. hot flushes and reflex tachycardia) compared to other dihydropyridine CCBs. Azelnidipine has been reported to reduce heart rate as opposed to inducing tachycardia. No evidence of bradycardia or complete atrioventricular block (CAVB) with azelnidipine treatment has been reported.

**Case presentation:**

In the present study, a 92-year-old woman was diagnosed with CAVB while taking azelnidipine and simvastatin for an extended period of time, and referred to our medical center. It was thought that the CAVB may have been an adverse effect of azelnidipine treatment. Specifically, it was considered that in this patient, one of the causes might be the concomitant use of simvastatin inhibiting the metabolism of azelnidipine by cytochrome P450 enzyme 3A4. Consequently, it was suggested to the patient’s physician that the patient’s serum azelnidipine levels be measured and treatment with azelnidipine and simvastatin be discontinued. The patient’s serum concentration of azelnidipine at the time of her visit to our center was 63.4 ng/mL, higher than the normal acceptable level. There was no occurrence of CAVB for 4 weeks, to present, following discontinuation of azelnidipine and simvastatin treatment.

**Conclusions:**

Azelnidipine has a different mechanism of action that other CCBs. In very rare cases, it may cause CAVB when combined with CYP3A4 inhibitors. If a patient taking azelnidipine is diagnosed with CAVB, physicians should suspect that the condition may be an adverse effect of azelnidipine and should consider discontinuing azelnidipine. And, in the elderly, it is necessary to avoid concomitant use of CYP3A4 inhibitors.

## Background

All calcium channel blockers (CCBs) commonly block the L-type calcium ion channel. The main therapeutic effects of CCBs include vasodilation of coronary and peripheral arteries, negative inotropic effects, and a decrease in heart rate and atrioventricular conduction [1, 2]. There are 3 categories of CCBs: dihydropyridines, benzothiazepines, and phenylalkylamines. Benzothiazepine CCBs (e.g., diltiazem) and phenylalkylamine CCBs (e.g., verapamil) are more selective for the myocardium than for vascular smooth muscle. Therefore, these CCBs are characterized by a weak antihypertensive effect and a strong negative inotropic effect, which may cause bradycardia and atrioventricular block. Dihydropyridine CCBs have strong vasodilator actions and are often used to treat hypertension. However, rapid vasodilation can cause adverse effect such as hot flushes, headache, and reflex tachycardia. Azelnidipine, classified as a dihydropyridine CCB, has a higher lipophilicity compared to other CCBs, resulting in higher affinity with vascular tissue and prolonged distribution in the tissue [3–5]. Therefore, its antihypertensive effects are mild, duration of its activities long, and a single dose daily can control blood pressure for 24 h [4, 6, 7]. In addition, azelnidipine has few adverse effects such as hot flushes and headaches. Whilst benzothiazepine and phenylalkylamine CCBs may result in bradycardia and atrioventricular block, dihydropyridine CCBs rarely cause such adverse effects, as shown in vivo [1, 2]. Rather, dihydropyridine CCBs generally trigger reflex tachycardia. Unlike other dihydropyridine CCBs, azelnidipine does not increase heart rate, but rather decreases it [4, 5, 7–9]. To the best of the authors’ knowledge, there have been no previous case reports of complete atrioventricular block (CAVB) with azelnidipine treatment. The present case report is one of an elderly patient who had been taking azelnidipine for a long time and had suspected CAVB, which she thought may be an adverse event associated with azelnidipine treatment.

## Case presentation

**Fig. 1:**
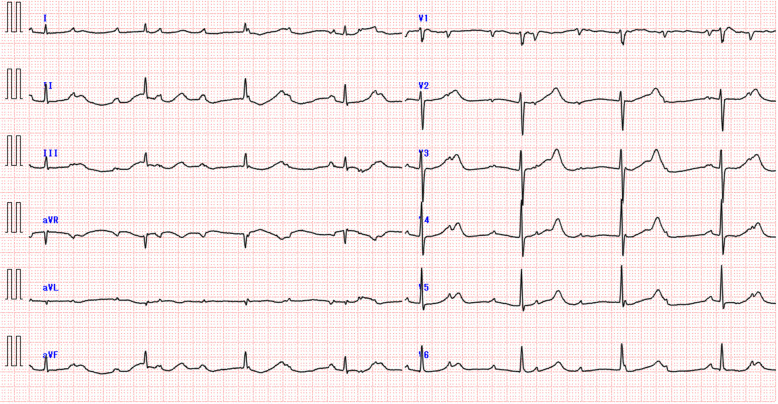
Electrocardiogram of patient on first arrival at the medical center

The patient was a 92-year-old woman who regularly saw her private physician and was being treated for hypertension, hyperlipidemia and chronic gastritis with azelnidipine 16 mg/day, simvastatin 5 mg/day, famotidine 20 mg/day, irsogladine maleate 4 mg/day and mosapride citrate hydrate 15 mg/day. Her medications had not been changed in over a decade, and she had never been diagnosed with CAVB. She complained of dyspnea and palpitation, and was consequently examined by her private physician (Day 1). She was prescribed furosemide 20 mg/day, spironolactone 25 mg/day and cibenzoline 100 mg/day. Three days later (Day 4), the patient was diagnosed with CAVB by electrocardiogram (ECG), and referred to our medical center by her private physician. When she arrived at our medical center, her blood pressure was 159/66 mmHg, and her heart rate was 44 beats/min. The patient’s ECG upon arrival at our center is shown in Fig. 1 and laboratory data is shown in Table 1. The patient was immediately hospitalized and underwent surgery for implantation of a temporary pacemaker, and intravenous furosemide treatment commenced. Oral administration of furosemide, spironolactone and cibenzoline was discontinued, however all other oral medications, including azelnidipine, continued. Three days after admission (Day 7), the patient returned to a normal sinus rhythm and the symptoms of dyspnea resolved. The temporary pacemaker was removed and intravenous furosemide discontinued. After removal of the temporary pacemaker, ECG examination did not show CAVB and the patient was discharged 9 days after her initial admission (Day 13). Three weeks after discharge from the hospital, she was again diagnosed by her private physician with CAVB and was again referred to our hospital (Day 34). As the patient had recurrent episodes of CAVB, it was arranged that she was registered for surgical implant of a permanent pacemaker. Her medications were investigated once again, and it was surmised that CAVB may have occurred as an adverse event associated with azelnidipine treatment. Both azelnidipine and simvastatin are metabolized by cytochrome P450 enzyme 3A4 (CYP3A4). These drugs are capable of competitively blocking the metabolism of the other. The authors proposed to the physician that azelnidipine and simvastatin be discontinued and the patient’s serum azelnidipine levels be measured, before surgery for the permanent pacemaker be conducted. The drugs were accordingly discontinued and surgical implant of a permanent pacemaker canceled. One week after discontinuation of the drugs (Day 41), a 24 h ambulatory ECG confirmed an episode of CAVB in the early morning, however no subjective symptoms such as dyspnea were observed. One month later a 24 h ambulatory ECG showed no episode of CAVB (Day 71). It was later observed that serum azelnidipine levels after 6 h of medication was 63.4 ng/mL. Over the course of the next 5 months, 24 h ambulatory ECGs were performed once a month, with no evidence of CAVB. The patient’s clinical course is summarized in Table 2.

**Table 1:** Laboratory data upon hospitalization

Laboratory parameter	Value	
White blood cell	12,100	count/μL
Red blood cell	351 × 10^4^	count/μL
Hemoglobin	11.0	g/dL
Platelet	24.6 × 10^4^	count/μL
Total protein	7.1	g/dL
Total albumin	3.7	g/dL
Total bilirubin	0.8	mg/dL
Total cholesterol	172	mg/dL
Cholinesterase	305	IU/L
Creatine Phosphokinase	94	IU/L
Aspartate aminotransferase	29	IU/L
Alanine aminotransferase	30	IU/L
Lactate dehydrogenase	254	IU/L
Blood urea nitrogen	48.9	mg/dL
Serum creatinine Serum creatinine	1.38	mg/dL
eGFR	27.5	mL/min/1.73 m^2^
Sodium	142	mEq/L
Potassium	4.8	mEq/L
Chlorine	109	mEq/L

*eGFR* Estimated glomerular filtration rate

**Table 2:** Clinical course of a 92-year-old female patient diagnosed with complete atrioventricular block

Time	Event
For more than 10 years before admission	Use of azelnidipine 16 mg/day and simvastatin 5 mg/day
Day 1	Patient complained of dyspnea and palpitations.
	Patient was prescribed furosemide, spironolactone and cibenzoline.
Day 4	Patient was diagnosed with complete atrioventricular block (CAVB) and hospitalized.
	Oral administration of furosemide, spironolactone and cibenzoline was discontinued.
	Intravenous administration of furosemide was started.
	Temporary pacemaker was implanted.
Day 7	Patient returned to a normal sinus rhythm, and the symptoms of dyspnea resolved.
	Intravenous administration of furosemide was discontinued.
	Temporary pacemaker was removed.
Day 13	Patient was discharged.
Day 34	Patient was again diagnosed with CAVB.
	Oral administration of azelnidipine and simvastatin was discontinued.
Day 41	Electrocardiogram (ECG) confirmed an episode of CAVB.
	No subjective symptoms were observed.
Day 71	ECG showed no episode of CAVB.
Over the course of the next 5 months	ECG was performed once a month, with no evidence of CAVB.

The Naranjo adverse drug reaction probability scale was used to determine the probability that CAVB was an adverse reaction associated with azelnidipine treatment [10]. The Naranjo scale generated a score of 5 (Table 3), suggesting a probable causal relationship between CAVB and azelnidipine treatment. A MEDLINE search was also conducted for articles published between 2003 and June 2021 with search terms such as “azelnidpine AND bradycardia” or “azelnidipine AND atrioventricular block”. However, the literature search did not result in any case studies reporting an association between azelnidipine and bradycardia or atrioventricular block.

**Table 3:** Naranjo Adverse Drug Reaction Probability Scale score of the patient

Questions	Answer	Score
Are there previous conclusive reports on this reaction?	No	0
Did the adverse event occur after the suspected drug was administered?	Yes	+ 2
Did the adverse ereaction improve when the drug was discontinued or a specific antagonist was administered?	Yes	+ 1
Did the adverse reaction reappear when the drug was readministered?	Do not know	0
Are there alternative causes (other than the drug) that could have on their own caused the reaction?	Do not know	0
Did the reaction reappear when a placebo was given?	Do not know	0
Was the drug detected in the blood (or other fluids) in concentrations know to be toxic?	Yes	+ 1
Was the reaction more severe when the dose was increased or less severe when the dose was decreased?	Do not know	0
Did the patient have a similar reaction to the same or similar drugs in any previous exposure?	Do not know	0
Was the adverse event confirmed by any objective evidence?	Yes	+ 1
Total score		5

## Discussion and conclusions

Dihydropyridine CCBs intrinsically reduce sinus node function and have a bradycardic effect, similar to benzothiazepine and phenylalkylamine CCBs. However, in vivo studies have shown that the sympathetic nervous system is stimulated by the antihypertensive reflex, which not only masks the intrinsic bradycardia effect, but also causes tachycardia when the reflex is strong [9]. An increase in heart rate has been shown to be an independent risk factor for cardiovascular events [11], indicating the importance of not elevating heart rate. Generally, at clinical doses, dihydropyridine CCBs do not prolong atrioventricular conduction or refractoriness or cause sinus node suppression [1]. However, azelnidipine, a dihydropyridine CCB, has been reported to cause a significant decrease in heart rate in dogs and spontaneously hypertensive rats (SHRs) [5, 9]. In clinical trials in humans, it has also been reported that long-term administration of azelnidipine tends to decrease heart rate [4, 6, 7]. These properties are thought to be attributed to the very small effect azelnidipine has on the sympathetic nervous system [4]. Therefore, azelnidipine differs from other dihydropyridine CCBs, in that it does not cause reflex tachycardia in vivo, but rather a decrease in heart rate. Kuramoto et al. reported a decrease in pulse rate of 2 beat/min after 6 weeks administration of azelnidipine at 16 mg/day [7], and Kario et al. reported a 3.5 beat/min decrease in pulse rate after 12 weeks of azelnidipine treatment [6]. No adverse events of bradycardia or atrioventricular block were reported. Katayama et al. reported that in a study of 210 hypertensive diabetic patients treated with azelnidipine 8 to 16 mg/day and temocapril hydrochloride 2 to 4 mg/day, respectively, there was only one case of possible atrioventricular block (AVB) that occurred as an adverse event due to treatment [12]. The authors noted however that the AVB case may have been an incidental adverse event.

Kuramoto et al. reported that Cmax and Tmax were 48.3 ± 19.0 ng/mL and 4.14 ± 1.46 h, respectively, in patients with essential hypertension treated daily with 16 mg azelnidipine for an extensive length of time [7]. In the present case report, the patient presented with a serum concentration of azelnidipine of 63.4 ng/mL 6 h after being administered 16 mg azelnidipine. The serum level of azelnidipine in the present case was well above the peak, even though the serum levels were measured well after Tmax. Therefore, we assume that if the serum levels of azelnidipine had been measured at Tmax, they would have been even higher than 63.4 ng/mL.

Our patient’s medications were managed by her family, so it was considered unlikely that she had overdosed on them. It is possible that the high serum levels in this case study are a result of a decrease in hepatic metabolic function due to age and is one of the causes of the drug’s interactions with CYP3A4. The metabolism of azelnidipine may have been competitively inhibited by combination treatment with simvastatin, which, similar to azelnidipine, is metabolized by CYP3A4. As most dihydropyridine CCBs are metabolized by CYP3A4, inhibition of CYP3A4 metabolization of CCBs may result in unchanged or high serum concentrations of dihydropyridine CCBs and subsequent adverse events [1, 2]. Common drugs with CYP3A4 inhibitory effects include itraconazole, cimetidine, and simvastatin [13]. Using human liver microsomes, Kazui et al. reported the Ki values of CYP3A4-inhibiting drugs against azelnidipine [14]. It was reported that metabolism of azelnidipine was inhibited by the CYP3A4-inhibiting drugs with the following Ki values: itraconazole, 0.065 μM; cimetidine, 0.8 mM, and simvastatin, 9.3 μM. As itraconazole strongly inhibits CYP3A4, concomitant use of it with azelnidipine is contraindicated [15]. The combination of simvastatin and azelnidipine treatment may also increase the serum levels of each drug and increase the risk of adverse effects associated with this combination treatment, as noted in the patient information booklet included in the combination treatment package [15]. The Cmax after oral administration of 5 mg simvastatin is 0.012 μM [16], which is well below the previously described Ki value of simvastatin (9.3 μM) [14]. Therefore, when simvastatin and azelnidipine are used concomitantly at normal doses, it is very unlikely that simvastatin will inhibit the metabolism of azelnidipine and consequently increase the blood concentration of the drug. However, as in the present case, there is a rare possibility that the concomitant use of these drugs may increase the blood concentration of azelnidipine, so caution should be exercised.

The patient in this case study had been taking azelnidipine and simvastatin for more than 10 years without any occurrence of CAVB. The authors conclude that the most likely cause for the sudden adverse event of CAVB is an age-related decrease in drug metabolism in the liver, however the reason for this remains unclear. In this patient, serum levels of azelnidipine at the time of CAVB occurrence were higher than normal, and indeed higher than reported peak levels. There was no occurrence of CAVB for 4 weeks, to present, following discontinuation of azelnidipine treatment. These results show that a score of 5 on the Naranjo scale is “probable”, indicating that CAVB is a possible adverse effect of azelnidipine treatment.

The findings of the present case study suggest that elderly patients receiving azelnidipine at 16 mg/day with concomitant CYP3A4 inhibitors may have elevated blood levels of azelnidipine, that may result in CAVB. Therefore, in the elderly, it is considered necessary to avoid concomitant use of CYP3A4 inhibitors.

## Data Availability

Data used in this case report will not be shared owing to the risk of identifying an individual.

## Abbreviations

AVB: Atrioventricular block
CAVB: Complete atrioventricular block
CCB: Calcium channel blocker
CYP3A4: Cytochrome P450 enzyme 3A4
ECG: Electrocardiogram
SHR: Spontaneously hypertensive rat

## References

[1] Abernethy DR, Schwart JB (1999). Calcium-antagonist drugs. N Engl J Med.

[2] McKeever RG, Hamilton RJ (2020). Calcium Channel Blockers: StatPearls [Internet].

[3] Wellington K, Scott LJ (2003). Azelnidipine. Drugs..

[4] Sada T, Saito H (2003). Pharmacological profiles and clinical effects of azelnidipine, a long-acting calcium channel blocker. Nihon Yakurigaku Zasshi.

[5] Sada T, Mizuno M, Miyama C, Ohata K, Oizumi K, Yamamura N (2002). Pharmacological properties of Azelnidipine, a long-acting Calcium Channel blocker with high-affinity for vascular tissues (part 2). Jpn Pharmacol Ther.

[6] Kario K, Sato Y, Shirayama M, Takahashi M, Shiosakai K, Hiramatsu K, Komiya M, Shimada K (2013). Inhibitory effects of azelnidipine tablets on morning hypertension. Drugs R D.

[7] Kuramoto K, Ichikawa S, Hirai A, Kanada S, Nakachi T, Ogihara T (2003). Azelnidipine and amlodipine: a comparison of their pharmacokinetics and effects on ambulatory blood pressure. Hypertens Res.

[8] Kumagaya H, Onami T, Iigaya K, Takimoto C, Hayashi K, Saruta T (2004). Mechanism of a reduction in heart rate by azelnidipine as investigated in terms of the peripheral and central nervous systems. Prog Med.

[9] Fujisawa M, Yorikane R, Chiba S, Koike H (2009). Chronotropic effect of azelnidipine, a slow- and long-acting dihydropyridine-type calcium channel blocker, in anesthetized dogs: a comparison with amlodipine. J Cardiovasc Pharmacol.

[10] Naranjo CA, Busto U, Sellers EM, Sandor P, Ruiz I, Roberts EA, Janecek E, Domecq C, Greenblatt DJ (1981). A method for estimating the probability of adverse drug reactions. Clin Pharmacol Ther.

[11] Palatini P, Julius S (1997). Heart rate and the cardiovascular risk. J Hypertens.

[12] Katayama S, Kawamori R, Iwamoto Y, Saito I, Kuramoto K (2008). In half of hypertensive diabetics, co-administration of a calcium channel blocker and an angiotensin-converting enzyme inhibitor achieved a target blood pressure of <130/80 mmHg: the azelnidipine and temocapril in hypertensive patients with type 2 diabetes (ATTEST) study. Hypertens Res.

[13] Ament PW, Bertolino JG, Liszewski JL (2000). Clinically significant drug interactions. Am Fam Physican.

[14] Kazui M, Ikeda T, Yamazoe Y (2004). Drug interaction of ca-channel blockers. Prog Med.

[15] Calblock (azelnidipine) [package insert]. Tokyo, Japan: Daiichi Sankyo Company, Ltd.; 2003.

[16] Lipovas (simvastatin) [interview form]. Tokyo, Japan: Organon; 2021.

